# Super high-resolution single-molecule sequence-based typing of HLA class I alleles in HIV-1 infected individuals in Ghana

**DOI:** 10.1371/journal.pone.0269390

**Published:** 2022-06-02

**Authors:** Nicholas I. Nii-Trebi, Saori Matsuoka, Ai Kawana-Tachikawa, Evelyn Y. Bonney, Christopher Z. Abana, Sampson B. Ofori, Taketoshi Mizutani, Aya Ishizaka, Teiichiro Shiino, Jun Ohashi, Taeko K. Naruse, Akinori Kimura, Hiroshi Kiyono, Koichi Ishikawa, William K. Ampofo, Tetsuro Matano

**Affiliations:** 1 AIDS Research Center, National Institute of Infectious Diseases, Tokyo, Japan; 2 Joint Research Center for Human Retrovirus Infection, Kumamoto University, Kumamoto, Japan; 3 Department of Medical Laboratory Sciences, School of Biomedical and Allied Health Sciences, University of Ghana, Accra, Ghana; 4 Institute of Medical Science, University of Tokyo, Tokyo, Japan; 5 Department of Virology, Noguchi Memorial Institute for Medical Research, University of Ghana, Accra, Ghana; 6 Department of Medicine, Koforidua Government Hospital, Eastern Region, Ghana; 7 Department of Biological Sciences, Graduate School of Sciences, University of Tokyo, Tokyo, Japan; 8 Department of Protozoology, Institute of Tropical Medicine, Nagasaki University, Nagasaki, Japan; 9 Department of Molecular Pathogenesis, Medical Research Institute, Tokyo Medical and Dental University, Tokyo, Japan; 10 Institute of Research, Tokyo Medical and Dental University, Tokyo, Japan; 11 Future Medicine Education and Research Organization, Chiba University, Chiba, Japan; 12 CU-UCSD Center for Mucosal Immunology, Allergy and Vaccines, Department of Medicine, University of California San Diego, San Diego, California, United States of America; University of California San Francisco, UNITED STATES

## Abstract

Polymorphisms in human leukocyte antigen (HLA) class I loci are known to have a great impact on disease progression in HIV-1 infection. Prevailing HIV-1 subtypes and HLA genotype distribution are different all over the world, and the HIV-1 and host HLA interaction could be specific to individual areas. Data on the HIV-1 and HLA interaction have been accumulated in HIV-1 subtype B- and C-predominant populations but not fully obtained in West Africa where HIV-1 subtype CRF02_AG is predominant. In the present study, to obtain accurate HLA typing data for analysis of HLA association with disease progression in HIV-1 infection in West African populations, HLA class I (*HLA-A*, *-B*, and *-C*) four-digit allele typing was performed in treatment-naïve HIV-1 infected individuals in Ghana (n = 324) by a super high-resolution single-molecule sequence-based typing (SS-SBT) using next-generation sequencing. Comparison of the SS-SBT-based data with those obtained by a conventional sequencing-based typing (SBT) revealed incorrect assignment of several alleles by SBT. Indeed, HLA-A*23:17, HLA-B*07:06, HLA-C*07:18, and HLA-C*18:02 whose allele frequencies were 2.5%, 0.9%, 4.3%, and 3.7%, respectively, were not determined by SBT. Several HLA alleles were associated with clinical markers, viral load and CD4^+^ T-cell count. Of note, the impact of HLA-B*57:*03* and HLA-B*58:*01*, known as protective alleles against HIV-1 subtype B and C infection, on clinical markers was not observed in our cohort. This study for the first time presents SS-SBT-based four-digit typing data on *HLA-A*, *-B*, and *-C* alleles in Ghana, describing impact of HLA on viral load and CD4 count in HIV-1 infection. Accumulation of these data would facilitate high-resolution HLA genotyping, contributing to our understanding of the HIV-1 and host HLA interaction in Ghana, West Africa.

## Introduction

One of the leading causes of mortality and morbidity in humans is infection [1]. Infectious diseases present a major selective pressure on the immune system [2]. Human leukocyte antigen (HLA) genes are important host genetic factors that play a fundamental role in immune recognition and response to foreign antigen. Association of HLA genotypes with susceptibility or resistance has been indicated in a variety of cancers and autoimmune disorders [3, 4] as well as infectious diseases [5–7].

HIV-1 infection remains to be a major global public health concern, having caused at least 35 million deaths over the past three decades with approximately 37 million people currently living with the virus [8]. Cytotoxic T-cell lymphocytes (CTLs) have been shown to play a critical role in controlling HIV-1 infection [9–12]. CTL responses exert strong suppressive pressure on HIV-1 replication and often select for viral genome mutations resulting in viral escape from CTL recognition [13, 14]. CTLs recognize viral antigen-derived epitope peptides bound to polymorphic HLA class I molecules, of which polymorphisms regulate CTL responses via determining binding antigenic peptide motifs. Thus, numbers of HLA-associated HIV-1 polymorphisms including CTL escape mutations have been reported [15]. Cumulative studies have indicated association of HLA class I alleles with viral load and disease progression in HIV-1 infection [7, 16–19]. For example, HLA-B*57, -B*58:*01* and -B*27, known as protective HLA alleles, associate with lower viral load and slow disease progression, whereas HLA-B*35 associates with higher viral load and rapid disease progression [20, 21].

Prevailing HIV-1 subtypes and HLA genotype distribution are different all over the world, and the HIV-1 and host HLA interaction could be specific to individual areas [15]. However, most of the data described above on HLA association with disease progression were derived from studies in Caucasian and Southern African populations, where HIV-1 subtype B or C is predominant. Data on the HIV-1 and HLA interaction have not fully been accumulated in West Africa including Ghana, where various non-subtype B/C, circulating and unique recombinant HIV-1 forms (CRF/URF) are prevailing [22–25].

It is important to collect accurate HLA genotyping data to evaluate the impact of host genetics on pathogenesis of various infectious diseases in African countries. Sequence based typing (SBT) has been developed and commercialized as a conventional HLA genotyping method [26, 27]. For SBT, the region including the exons 2 and 3 of *HLA-A*, *-B*, and *-C* are amplified by polymerase chain reaction (PCR) and sequenced directly by Sanger method. However, the SBT often detects more than one pair of unresolved HLA alleles because of chromosomal phase ambiguity [28], and assignment of the HLA alleles is carried out by a predicted selection of the most frequent HLA alleles in the population according to the existed HLA allele frequency data. It is almost impossible to accurately assign HLA alleles by SBT in African populations with high diversity but very limited data on HLA allele frequencies. Recently, the super high-resolution single-molecule sequence-based typing (SS-SBT) has been developed using next generation sequencing (NGS) [29–31]. The SS-SBT could resolve the issue of phase ambiguities and allow the assignment to single HLA allele and an efficient detection of new HLA alleles.

In the present study, to obtain accurate HLA typing data for analysis of HLA association with HIV-1 disease progression in West African populations, HLA class I (*HLA-A*, *-B*, and *-C*) allele typing was performed by SS-SBT in HIV-1-infected individuals in Ghana. We then examined association of *HLA* alleles with plasma viral load and CD4 count, strong predictors of disease progression, in treatment-naïve HIV-1-infected individuals.

## Materials and methods

### Ethics statement

This study was approved by the Institutional Review Board of Noguchi Memorial Institute for Medical Research (NMIMR), University of Ghana, Ghana (approval number: 071/11-12) and the Ethical Committee of the National Institute of Infectious Diseases (NIID), Tokyo, Japan (Approval number: 351). The written informed consent was provided by all the participants.

### Study population, sample collection and examination of clinical markers

Antiretroviral therapy (ART)-naïve individuals infected with HIV-1 were studied. Study participants were recruited through voluntary counselling and testing at the Koforidua Government hospital in the Eastern Region of Ghana from 2013 to 2014 (estimated sample size was approximately 300). HIV-1 and HIV-2 infection was confirmed by (INNO-LIA HIV I/II Score, INNOGENETICS N.V.) after recruitment to this study. Whole blood samples were collected from a cohort of 353 (pre-ART) individuals at the recruitment at the Koforidua Government hospital, while the seroconversion date was unclear. Peripheral blood mononuclear cells (PBMCs) and plasma were isolated from whole blood by Ficoll-Hypaque (Sigma-Aldrich). Plasma HIV-1 viral load (VL) and peripheral CD4 count were measured by the Roche Amplicor Monitor version 1.5 assay (Roche Applied Science) and FACSCount flow cytometer (Beckman Coulter Ltd.), respectively.

Out of the 353 participants, four were confirmed to be HIV-negative and eleven were HIV-1-negative/HIV-2-positive. In the remaining 338 HIV-1-positive individuals, we attempted to determine HLA class I alleles by SS-SBT. *HLA-A*, *-B and -C* alleles were successfully determined by SS-SBT in 324 of the 338 individuals and were further analyzed in the present study. Viral *gag* sequences were determined in 246 of the 324 individuals in our previous study [32] showing that 200 (81.3%) were infected with HIV-1 subtype CRF02_AG. The remaining samples consisted of CRF06_cpx (*n* = 14), CRF09_cpx (*n* = 4), CRF11_cpx (*n* = 23), and CRF13_cpx/CRF14_BG (*n* = 5) as described previously [32].

Demographic and clinical characteristics of the 324 individuals are shown in Table 1. All 324 individuals were ART naive. They were multi-lingual, consisting mainly of Akans, Ewes, Krobos, Gas and Hausas. Of the 324 individuals, 285 (88.0%) were infected via heterosexual transmission, and 27 (8.3%) were both HIV-1 and HIV-2 positive. Their median CD4^+^ T cell counts and plasma viral loads were 325 cells/μl (IQR 141 to 526) and 5.18 log_10_ copies/ml (IQR 4.52 to 5.77), respectively, as described before [32]. No significant difference in CD4^+^ T cell counts (cells/μl) (*p* = 0.5354 by Mann-Whitney U test) was observed between in HIV-1-positive/HIV-2-negative (n = 297; median 326; IQR 137 to 527) and in HIV-1-positive/HIV-2-positive (n = 27; median 295; IQR 237 to 521). No significant difference in plasma viral loads (log_10_ copies/ml) (*p* = 0.6013 by Mann-Whitney U test) was observed between in HIV-1-positive/HIV-2-negative (n = 297; median 5.17; IQR 4.53 to 5.76) and in HIV-1-positive/HIV-2-positive (n = 27; median 5.24; IQR 4.33 to 5.85), either.

**Table 1:** Clinical information of samples (n = 324)^a^.

Characteristic		n (%)	Median (IQR)
Age (years)			40 (33–48)
Gender	Female	233 (71.9%)	
	Male	91 (28.1%)	
Transmission route	Heterosexual	285 (88.0%)	
	Blood transfusion	1 (0.3%)	
	Unknown	34 (10.5%)	
	Other	4 (1.2%)	
HIV serology	HIV-1(+)/HIV-2(-)	297 (91.7%)	
	HIV-1(+)/HIV-2(+)	27 (8.3%)	
CD4 count (cells/μl)			325 (141–526)
	>500	92 (28.4%)	
	201–500	119 (36.7%)	
	50–200	68 (21.0%)	
	<50	45 (13.9%)	
Viral load (copies/ml)^b^			5.18 (4.52–5.77)
	>10^6^	49 (15.1%)	
	10^3^–10^6^	260 (80.2%)	
	20–10^3^	7 (2.2%)	
	<20	8 (2.5%)	

^a^Data on Age (2 of the 324 were unknown), Gender, Median and IQR of CD4 count and Viral load were described previously [32] and used under a CC BY license.

^b^The lower limit of detection is 20 copies/ml.

### HLA class I genotyping

*HLA-A*, *-B and -C* genotyping was performed by SS-SBT and SBT. Total cellular DNAs were extracted from PBMCs using the QIAamp DNA Blood Mini Kit (Qiagen), and used for the HLA class I genotyping. The SS-SBT was performed by Geno Dive Pharma (Kanagawa, Japan) as described before [29–31]. Briefly, entire gene regions from the promoter-enhancer region to the 3’UTR of *HLA-A*, *-B*, and *-C* were independently amplified by a previously developed long-range PCR method, and sequence information was obtained using the GS Junior System (Roche). Assignment of 4-digit alleles was performed in reference to NGSengine ver.2.5.1.8622 and HLATypeStream ver. 1.1.0.11 software. The SBT was carried out by nested PCR amplification and direct sequencing of regions spanning exons 2 and 3 of *HLA-A*, *-B and -C* loci, using universal, locus-specific primers as described before [27]. Sequencing data were acquired in ABI 3500XL Genetic Analyzer (Applied Biosystems). Four-digit alleles were determined by using GenDX SBTengine software ver. 3.19.0 (GenDX), which assigns alleles by comparison of test samples with reference standards reported in the ImMunoGeneTics (IMGT)/HLA Database [33].

### Statistical analysis

Deviation of genotypic frequencies from the Hardy–Weinberg equilibrium (HWE) at each *HLA* locus was tested by Genepop version 4.2 web project [34, 35]. The exact *p*-value of this test was estimated by a Markov chain algorithm [36] with 10,000, 1,000, and 10,000 as the dememorization number, the number of batches, and the number of iterations per batch, respectively. The association analyses were performed for 61 *HLA* alleles whose population frequency was higher than 1% in 324 subjects. In the association analyses, the log-transformed data (i.e., log_10_[VL] and log_10_[CD4 count]) were compared between subjects expressing the relevant alleles and those not expressing that allele by Welch’s t-test using R software. In this study, the *p*-value of less than 0.05 was regarded as statistically significant. The corrected *p*-value was also calculated for *HLA* allele associated with VL or CD4 count. To obtain the corrected *p*-value, the *p*-value of the associated *HLA* allele was multiplied by the number of *HLA* alleles tested at the relevant *HLA* locus.

## Results

### *HLA-A*, *-B*, and *-C* genotyping by SS-SBT

**Fig 1:**
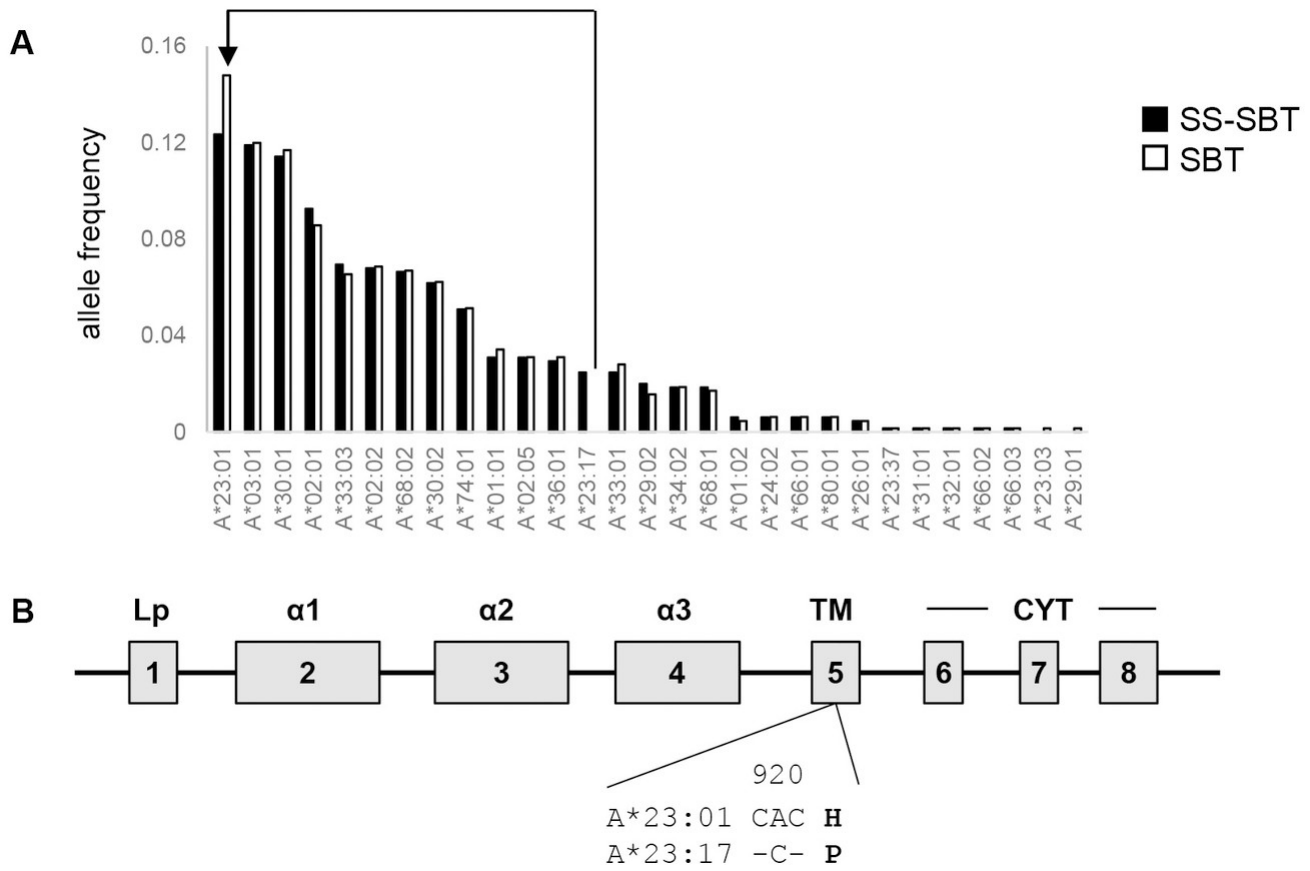
*HLA-A* allele frequencies in HIV-1 infected individuals in Ghana. A. *HLA-A* allele frequencies assigned by SS-SBT (filled) and SBT (open) are shown. An HLA allele pair showing discrepancy between the typing methods (A*23:*17* by SS-SBT and A*23:*01* by SBT) is shown by an arrow-line. B. Schema of HLA class I gene structure. The difference in amino acid sequences between A*23:*01* and A*23:*17* is shown.

**Fig 2:**
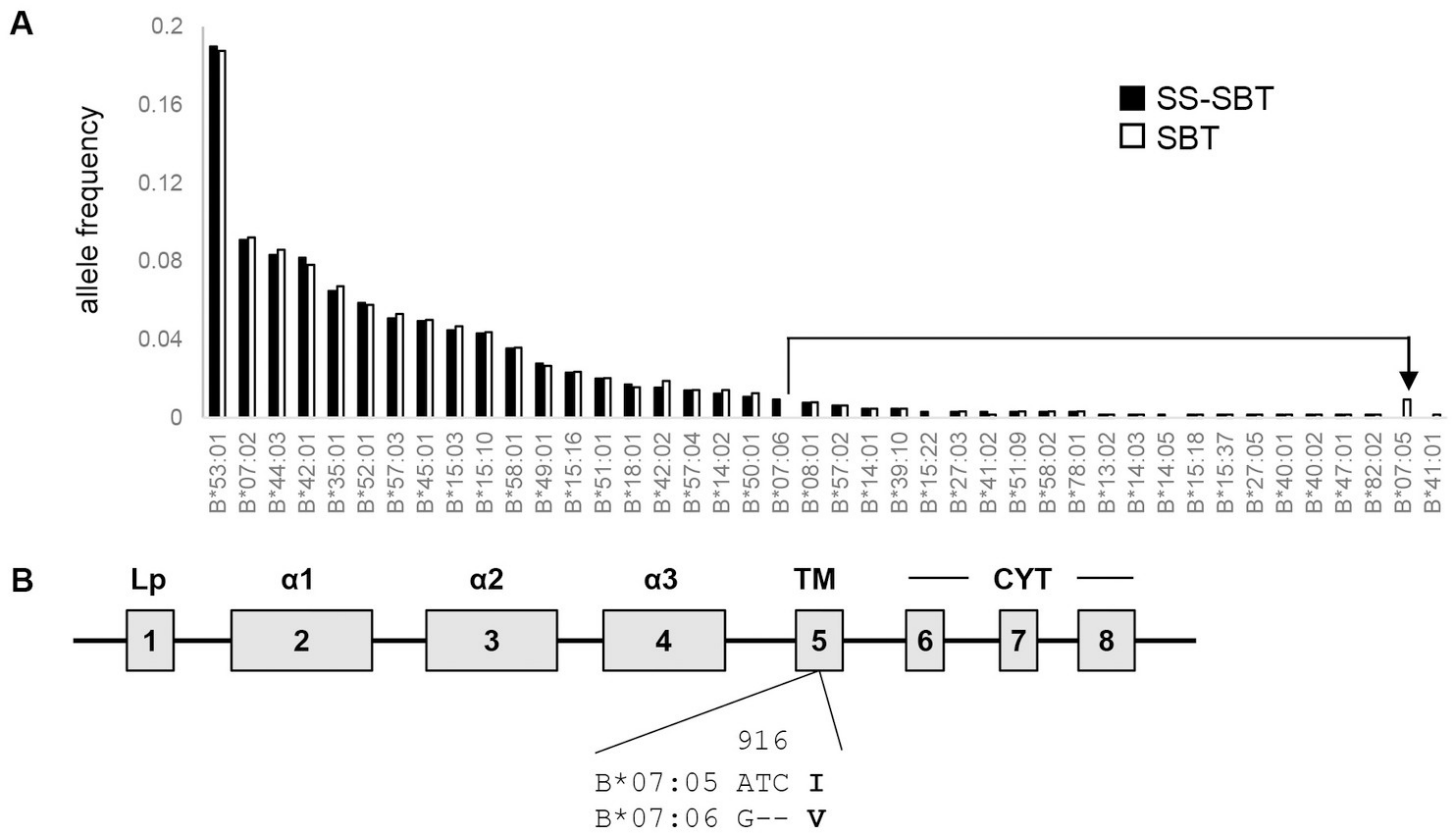
*HLA-B* allele frequencies in HIV-1 infected individuals in Ghana. A. *HLA-B* allele frequencies assigned by SS-SBT (filled) and SBT (open) are shown. An HLA allele pair showing discrepancy between the typing methods (B*07:*06* by SS-SBT and B*07:*05* by SBT) is shown by an arrow-line. B. Schema of HLA class I gene structure. The difference in amino acid sequences between B*07:*05* and B*07:*06* is shown.

**Fig 3:**
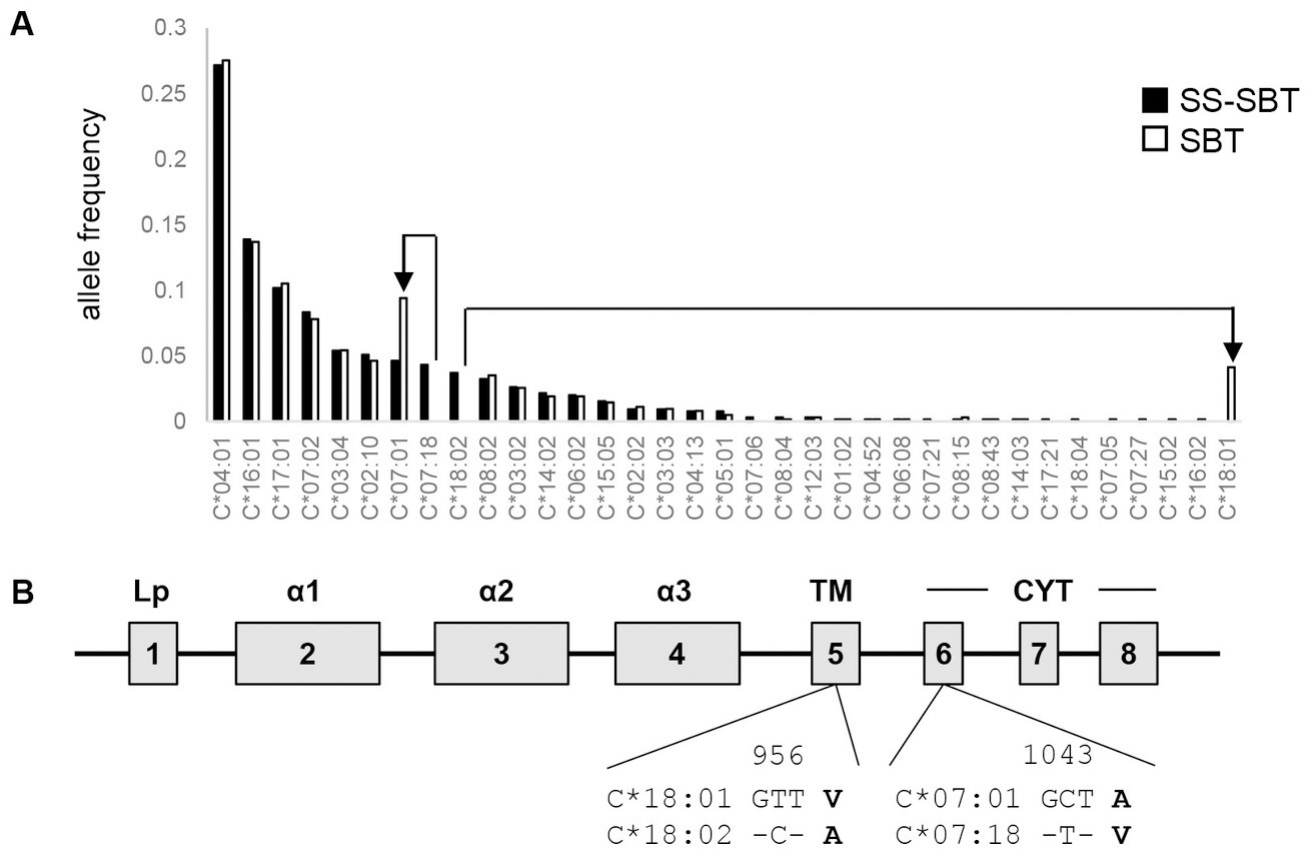
*HLA-C* allele frequencies in HIV-1 infected individuals in Ghana. A. *HLA-C* allele frequencies assigned by SS-SBT (filled) and SBT (open) are shown. HLA allele pairs showing discrepancy between the typing methods (C*07:*18* by SS-SBT and B*07:*01* by SBT; C*18:*02* by SS-SBT and B*18:*01* by SBT) are shown by arrow-lines. B. Schema of HLA class I gene structure. The differences in amino acid sequences between C*07:*01* and C*07:*18* and between C*18:*01* and C*18:*02* are shown.

Study participants (n = 353) were recruited at the Koforidua Government hospital in the Eastern Region of Ghana. Of the 353 participants, 338 were confirmed to be HIV-1-positive. *HLA-A*, *-B*, and *-C* genotyping was performed by SS-SBT with long-range PCR, and 4-digit alleles were assigned in this study. *HLA-A*, *-B and -C* alleles were successfully determined by SS-SBT in 324 of the 338 individuals. Twenty-seven, 40, and 30 alleles were found in *HLA-A*, *-B*, and *-C* loci, respectively (Figs 1–3, S1–S3 Tables). Predominantly observed alleles whose frequencies were higher than 10% were A*23:*01*, A*03:*01*, and A*30:*01* at the *HLA-A* locus, B*53:*01* at the *HLA-B* locus, and C*04:*01*, C*16:*01*, and C*17:*01* at the *HLA-C* locus.

### Comparison of HLA genotyping data obtained by SS-SBT and by SBT

HLA genotyping was also performed by a conventional SBT. SBT failed to assign *HLA-A*, *-B*, and *-C* alleles in 3, 2, and 10 of the 324 samples, respectively, because of the low quality of the sequences. Comparison between SBT- and SS-SBT-based HLA genotyping data showed several discrepancies. In the *HLA-A* locus, A*23:*17* was assigned by SS-SBT in 16 samples (allele frequency: 2.5%), while these were assigned as A*23:*01* by SBT (Fig 1A). In the *HLA-B* locus, B*07:*06* was assigned by SS-SBT in 6 samples (allele frequency: 0.9%), while these were assigned as B*07:*05* by SBT (Fig 2A). In the *HLA-C* locus, C*07:*18* and C*18:*02* were assigned by SS-SBT in 28 and 24 samples (allele frequency: 4.3% and 3.7%), respectively, while these were assigned as C*07:*01* and C*18:*01* by SBT, respectively (Fig 3A). These mismatched alleles have only one nucleotide difference, and all of the polymorphisms were located in exons 5 and 6, out of the region for sequencing in SBT (Figs 1B, 2B and 3B). These results revealed inaccuracy of SBT-based HLA genotyping, indicating that SS-SBT is currently required for accurate HLA genotyping in Ghana.

The observed genotype frequencies determined by SS-SBT were significantly deviated from those expected from HWE at the *HLA-B* locus (*p*-value = 0.0122), but not at *HLA-A* (*p*-value = 0.7871) and *-C* (*p*-value = 0.5689). It is unlikely that genotyping errors caused the significant deviation. There may be specific *HLA-B* alleles or genotypes associated with HIV-1 susceptibility or HIV-1-related symptoms resulting in visit to the hospital. Alternatively, there may be the Wahlund effect, since different ethnic subpopulations were included. Examination of these possibilities including comparison of *HLA-B* allele frequencies between HIV-1 positive and HIV-1 negative subjects might be the next issue to be determined.

### Association of HLA genotypes with viral loads and CD4 counts in HIV-1 infected individuals in Ghana

**Fig 4:**
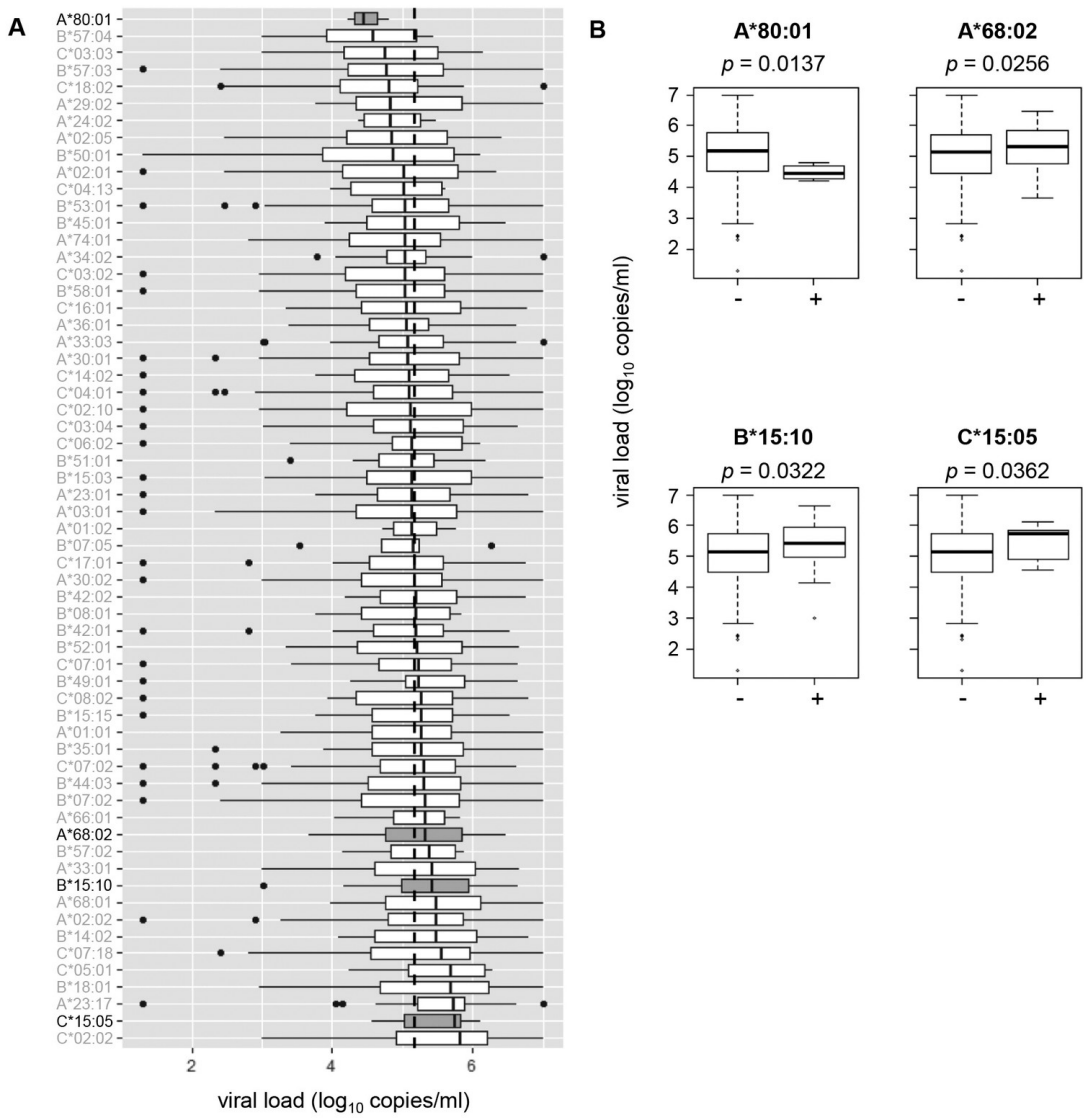
Association between HLA class I allele expression and viral loads. A. Association of individual HLA class I alleles (61 alleles with higher than 1% population frequencies) with viral loads. Vertical dotted line indicates cohort median viral load. Shading box indicates *p* < 0.05. B. Comparison of viral loads between individuals with and without each HLA allele that showed a significant association in A.

**Fig 5:**
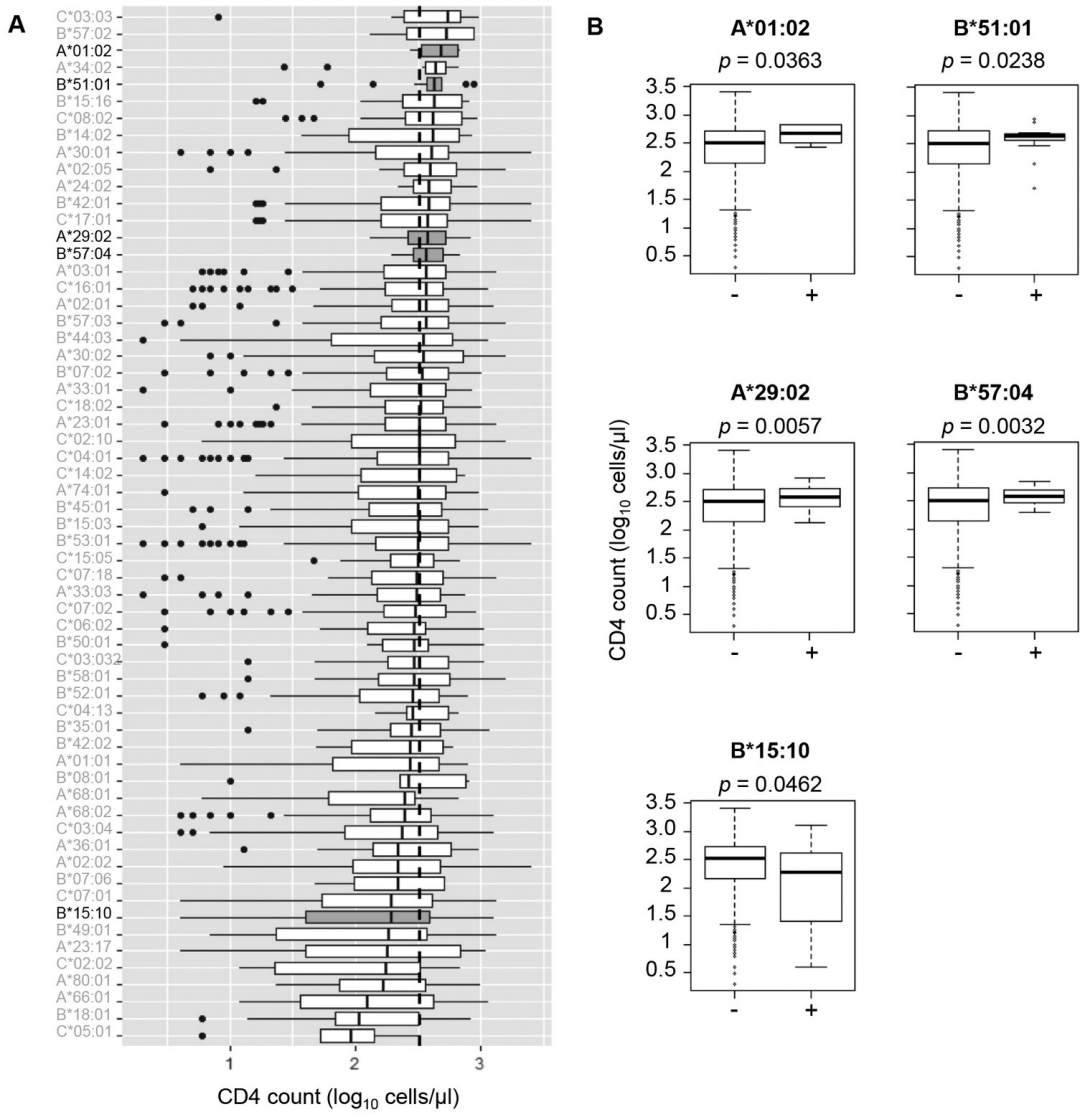
Association between HLA class I allele expression and CD4 counts. A. Association of individual HLA class I alleles (61 alleles with higher than 1% population frequencies) with CD4 counts. Vertical dotted line indicates cohort median CD4 count. Shading box indicates *p* < 0.05. B. Comparison of CD4 counts between individuals with and without each HLA allele that showed a significant association in A.

We then examined the influence of individual HLA alleles on viral loads and CD4 counts in our Ghanaian cohort. Sixty-one HLA alleles with higher than 1% population frequencies were assessed. A*80:*01* was significantly associated with lower viral loads, and A*68:*02*, B*15:*10*, and C*15:*05* were significantly associated with higher viral loads (Fig 4). A*01:*02*, B*51:*01*, A*29:*02*, and B*57:*04* were significantly associated with higher CD4 counts, whereas B*15:*10* were significantly associated with lower CD4 counts (Fig 5). Although none of the *HLA* alleles showed the corrected *p*-values less than 0.05 (S4 Table), B*15:*10* was associated with both higher viral loads and lower CD4 counts, which strongly suggests that this allele may be detrimental for HIV-1 control. While B*57:*03* and B*58:*01* are well-known protective alleles in several study cohorts of HIV-1 subtypes B and C infected subjects [15, 20, 37–39], these alleles showed no significant influence on viral load or CD4 count in this cohort.

## Discussion

Cumulative studies have described the great impact of HLA genotypes on HIV-1 infection [7, 13, 15–21]. Most of the findings were based on the analysis of HIV-1 subtype B or C infection, while HLA genotypes may have different impact on infections of HIV-1 subtypes other than subtypes B and C. We then investigated HLA class I genotypes in HIV-1-infected individuals in Ghana, West Africa, where HIV-1 subtype CRF02_AG is predominant.

The strength and credibility of the HLA class I typing data reported here lie in the use of powerful technique for HLA genotyping using NGS. In SBT that has been mostly used as a high-resolution HLA genotyping, direct nucleotide sequencing of PCR amplicon spanning exon 2 to exon 3 of individual *HLA* loci is performed, and then, HLA genotypes are assigned based on pre-existed HLA allele frequency information [26, 27]. Therefore, it is difficult to perform accurate assignment of HLA genotypes by SBT in the populations including Ghana, where data on HLA allele frequency have not been accumulated. Furthermore, ambiguity resulting from the *cis*/*trans* assignment of base calls in heterozygous samples makes allele determination difficult. In contrast, these limitations in SBT can be overcome in SS-SBT, which utilizes the entire HLA gene sequence from a single DNA molecule [29–31]. In the present study, SS-SBT enabled us to find four HLA alleles, A*23:*17*, B*07:*06*, C*07:*18*, and C*18:*02*, which have polymorphisms in HLA exon 5 or 6, in Ghana. These four alleles, whose frequencies were higher than 0.9% in our cohort, were not detected by SBT. Although amino acid sequences of antigen binding domain encoded in the exons 2 and 3 of these alleles are identical to A*23:01, C*07:05, C*07:01, C*18:01, respectively, amino acid changes in the exons 5 and 6 that encode the HLA transmembrane region and cytoplasmic tail may affect the stability of the HLA molecule on the cell surface. The influence of surface expression levels of HLA molecules on HIV control has been reported [40], indicating the significance of our HLA genotype data obtained by SS-SBT for analysis of its association with HIV control. This study provides accurate HLA class I four-digit allele data in Ghana, which could help improvement of accuracy of SBT-based HLA determination in Ghana.

By using these HLA allele data obtained by SS-SBT, this study describes data on association analyses between HLA alleles and viral loads or CD4 counts in HIV-1 infected individuals in Ghana. Several alleles were shown to be associated with higher or lower viral loads or CD4 counts. In particular, association of HLA-B*15:*10* whose serological type is equivalent to B*71 [41, 42] with both higher viral loads and lower CD4 counts suggests that this allele may be detrimental for HIV-1 control. However, none of the alleles showed corrected *p*-value of less than 0.05, suggesting that the impact of these HLA class I alleles on disease progression is not large in our cohort. Multivariate regression analysis with correction for age and sex showed no significant association between HLA alleles and viral loads or CD4 counts, either. In this study, to avoid false negatives due to multiple testing corrections by conservative methods (e.g., Bonferroni correction or corrected *p*-value), the statistical significance was considered based on the *p*-value obtained by Welch’s t-test. Indeed, multiple testing corrections sometimes cause false-negative results. In a previous report, HLA-B*81:*01* was significantly associated with lower viral loads before Bonferroni correction, but the significance was lost after the correction [20]. However, the association of HLA-B*81:*01* with lower viral loads has been confirmed in multiple reports [40, 43] and the HLA-B*81:*01* is now recognized as a protective allele. We thus consider that the results without any correction should be taken into account for interpretation, while the corrected *p*-values were also calculated for association analyses.

HLA-B*35:*01* is relatively predominant at the *HLA-B* locus in West African population [15] including our cohort. In Caucasians and African-Americans, strong association of HLA-B*35:*01* with higher viral loads and accelerated disease progression has been indicated [44]. However, a recent study has shown differential clade-specific B*35:*01* association with HIV-1 disease outcome, and association of B*35:*01* with higher viral loads was not observed in HIV-1 clade C-infected African cohorts [45]. In our Ghanaian cohort mostly infected with HIV-1 subtype CRF02_AG, significant association of HLA-B*35:*01* with higher viral loads or lower CD4 counts was not observed, either.

HLA-B*57:*03* and HLA-B*58:*01* are known as protective alleles strongly correlated with lower viral loads in HIV-1 subtype B and C infections [15]. In our cohort, allele frequencies of HLA-B*57:*03* and HLA-B*58:*01* are 5.1% and 3.6%, respectively, but no association of these alleles with viral loads or CD4 counts was observed. The mechanism for this discrepancy in the impact of these alleles on clinical markers between HIV-1 subtype B/C predominant populations and our cohort would be a next issue to be determined, but difference in prevailing viral genome sequences may be involved in this discrepancy. Indeed, differences in amino acid sequences in B*57/B*58:01-restricted Gag CTL epitopes and the flanking regions are observed between HIV-1 subtype B/C and CRF02_AG [46].

HLA class I molecules are the ligands for the killer cell immunoglobulin-like receptors (KIR) expressed on NK cells, and the interaction between HLA class I and KIR modulates NK cell activity. It has been reported that the specific combination of KIRs and HLA alleles is associated with HIV disease progression [47]. KIR typing was not performed in this study, but may contribute to analysis of possible association between HLA-KIR genotypes and the clinical parameters in HIV-1 infection.

This study has some limitations. First, the sample size was small and hence may not afford sufficient statistical power to detect all really significant associations. Second, our cohort was consisting of multiple ethnic subpopulations, which may have some effect on association analyses. Further comparable population studies, particularly in CRF02_AG-infected cohorts, would contribute to corroboration of our findings.

In summary, this study for the first time presents SS-SBT-based four-digit typing data on *HLA-A*, *-B*, and *-C* alleles in Ghana. Analysis using the typing data indicated association of several HLA class I alleles on viral load an CD4 count in HIV-1 infection. Accumulation of these data would facilitate high-resolution HLA genotyping, contributing to our understanding of the HIV-1 and host HLA interaction in Ghana, West Africa.

## Supporting information

S1 Table HLA-A allele frequencies in HIV-1 infected individuals in Ghana. (PDF)

S2 Table HLA-B Allele frequencies in HIV-1 infected individuals in Ghana. (PDF)

S3 Table HLA-C allele frequencies in HIV-1 infected individuals in Ghana. (PDF)

S4 Table Association of HLA-A, -B, and -C alleles with CD4 counts and viral loads. (PDF)
