# Supplementary material for: Super high-resolution single-molecule sequence-based typing of HLA class I alleles in HIV-1 infected individuals in Ghana
Source: PLoS One. 2022 Jun 2;17(6):e0269390. doi: 10.1371/journal.pone.0269390 (PMC9162337; doi:10.1371/journal.pone.0269390)
Supplement: S1 Table — (PDF) [file pone.0269390.s001.pdf]

S1 Table. HLA-A allele frequencies in HIV-1 infected individuals in Ghana[a]

allele	n	allele frequency
A*01:01	20	0.0309
A*01:02	4	0.0062
A*02:01	60	0.0926
A*02:02	44	0.0679
A*02:05	20	0.0309
A*03:01	77	0.1188
A*23:01	80	0.1235
A*23:17	16	0.0247
A*23:37	1	0.0015
A*24:02	4	0.0062
A*26:01	3	0.0046
A*29:02	13	0.0201
A*30:01	74	0.1142
A*30:02	40	0.0617
A*31:01	1	0.0015
A*32:01	1	0.0015
A*33:01	16	0.0247
A*33:03	45	0.0694
A*34:02	12	0.0185
A*36:01	19	0.0293
A*66:01	4	0.0062
A*66:02	1	0.0015
A*66:03	1	0.0015
A*68:01	12	0.0185
A*68:02	43	0.0664
A*74:01	33	0.0509
A*80:01	4	0.0062
total	648	1.0000

[a]Alleles whose frequency is higher than 0.1 (10%) are highlighted.
