# Supplementary material for: Super high-resolution single-molecule sequence-based typing of HLA class I alleles in HIV-1 infected individuals in Ghana
Source: PLoS One. 2022 Jun 2;17(6):e0269390. doi: 10.1371/journal.pone.0269390 (PMC9162337; doi:10.1371/journal.pone.0269390)
Supplement: S2 Table — (PDF) [file pone.0269390.s002.pdf]

S2 Table. HLA-B Allele frequencies in HIV-1 infected individuals in Ghana[a]

allele	n	allele frequency
B*07:02	59	0.0910
B*07:06	6	0.0093
B*08:01	5	0.0077
B*13:02	1	0.0015
B*14:01	3	0.0046
B*14:02	8	0.0123
B*14:03	1	0.0015
B*14:05	1	0.0015
B*15:03	29	0.0448
B*15:10	28	0.0432
B*15:16	15	0.0231
B*15:18	1	0.0015
B*15:22	2	0.0031
B*15:37	1	0.0015
B*18:01	11	0.0170
B*27:03	2	0.0031
B*27:05	1	0.0015
B*35:01	42	0.0648
B*39:10	3	0.0046
B*40:01	1	0.0015
B*40:02	1	0.0015
B*41:02	2	0.0031
B*42:01	53	0.0818
B*42:02	10	0.0154
B*44:03	54	0.0833
B*45:01	32	0.0494
B*47:01	1	0.0015
B*49:01	18	0.0278
B*50:01	7	0.0108
B*51:01	13	0.0201
B*51:09	2	0.0031
B*52:01	38	0.0586
B*53:01	123	0.1898
B*57:02	4	0.0062
B*57:03	33	0.0509
B*57:04	9	0.0139
B*58:01	23	0.0355
B*58:02	2	0.0031
B*78:01	2	0.0031
B*82:02	1	0.0015
total	648	1.0000

[a]Alleles whose frequency is higher than 0.1 (10%) are highlighted.
