# Supplementary material for: Super high-resolution single-molecule sequence-based typing of HLA class I alleles in HIV-1 infected individuals in Ghana
Source: PLoS One. 2022 Jun 2;17(6):e0269390. doi: 10.1371/journal.pone.0269390 (PMC9162337; doi:10.1371/journal.pone.0269390)
Supplement: S3 Table — (PDF) [file pone.0269390.s003.pdf]

S3 Table. HLA-C allele frequencies in HIV-1 infected individuals in Ghana[a]

allele	n	allele frequency
C*01:02	1	0.0015
C*02:02	6	0.0093
C*02:10	33	0.0509
C*03:02	17	0.0262
C*03:03	6	0.0093
C*03:04	35	0.0540
C*04:01	176	0.2716
C*04:13	5	0.0077
C*04:52	1	0.0015
C*05:01	5	0.0077
C*06:02	13	0.0201
C*06:08	1	0.0015
C*07:01	30	0.0463
C*07:02	54	0.0833
C*07:06	2	0.0031
C*07:18	28	0.0432
C*07:21	1	0.0015
C*08:02	21	0.0324
C*08:04	2	0.0031
C*08:15	1	0.0015
C*08:43	1	0.0015
C*12:03	2	0.0031
C*14:02	14	0.0216
C*14:03	1	0.0015
C*15:05	10	0.0154
C*16:01	90	0.1389
C*17:01	66	0.1019
C*17:21	1	0.0015
C*18:02	24	0.0370
C*18:04	1	0.0015
total	648	1.0000

[a]Alleles whose frequency is higher than 0.1 (10%) are highlighted.
