# Supplementary material for: Super high-resolution single-molecule sequence-based typing of HLA class I alleles in HIV-1 infected individuals in Ghana
Source: PLoS One. 2022 Jun 2;17(6):e0269390. doi: 10.1371/journal.pone.0269390 (PMC9162337; doi:10.1371/journal.pone.0269390)
Supplement: S4 Table — (PDF) [file pone.0269390.s004.pdf]

S4 Table. Association of HLA-A, -B, and -C alleles with CD4 counts and viral loads[a]

Allele	Positivity	CD4 count		Viral load	
		P	$P_{corrected}$	*P*	$P_{corrected}$
A*01:01	0.0617	0.3038	-	0.4884	-
A*01:02	0.0123	**0.0363***	0.7623	0.5682	-
A*02:01	0.1728	0.1794	-	0.1519	-
A*02:02	0.1358	0.5210	-	0.2817	-
A*02:05	0.0617	0.2061	-	0.3445	-
A*03:01	0.2130	0.6434	-	0.2531	-
A*23:01	0.2315	0.9733	-	0.8624	-
A*23:17	0.0494	0.2279	-	0.3828	-
A*24:02	0.0123	0.1136	-	0.5860	-
A*29:02	0.0401	**0.0057***	0.1201	0.8656	-
A*30:01	0.2130	0.1864	-	0.8115	-
A*30:02	0.1204	0.8134	-	0.4181	-
A*33:01	0.0494	0.7059	-	0.5408	-
A*33:03	0.1389	0.7166	-	0.4731	-
A*34:02	0.0370	0.2035	-	0.8411	-
A*36:01	0.0586	0.9784	-	0.7682	-
A*66:01	0.0123	0.5978	-	0.8318	-
A*68:01	0.0370	0.1599	-	0.1353	-
A*68:02	0.1327	0.2487	-	**0.0256***	0.5367
A*74:01	0.1019	0.4885	-	0.3816	-
A*80:01	0.0123	0.7175	-	**0.0137***	0.2872
B*07:02	0.1790	0.6380	-	0.7244	-
B*07:06	0.0154	0.8333	-	0.8864	-
B*08:01	0.0154	0.9548	-	0.8679	-
B*14:02	0.0247	0.7559	-	0.3353	-
B*15:03	0.0895	0.8966	-	0.9220	-
B*15:10	0.0864	**0.0462***	1.0173	**0.0322***	0.7089
B*15:16	0.0463	0.3579	-	0.7668	-
B*18:01	0.0340	0.1367	-	0.3805	-
B*35:01	0.1235	0.0949	-	0.4224	-
B*42:01	0.1605	0.1280	-	0.4996	-
B*42:02	0.0309	0.9491	-	0.3431	-
B*44:03	0.1574	0.3029	-	0.7332	-
B*45:01	0.0895	0.6617	-	0.8069	-
B*49:01	0.0556	0.1414	-	0.4499	-
B*50:01	0.0216	0.7270	-	0.4171	-
B*51:01	0.0401	**0.0238***	0.5230	0.9302	-
B*52:01	0.1142	0.5851	-	0.7252	-
B*53:01	0.3426	0.8342	-	0.7085	-
B*57:02	0.0123	0.2320	-	0.7145	-
B*57:03	0.1019	0.7982	-	0.2046	-
B*57:04	0.0278	**0.0032***	0.0708	0.0679	-
B*58:01	0.0710	0.2938	-	0.4714	-
C*02:02	0.0185	0.3326	-	0.5301	-
C*02:10	0.1019	0.8165	-	0.8191	-
C*03:02	0.0525	0.6567	-	0.5026	-

C*03:03	0.0185	0.8054	-	0.5241	-
C*03:04	0.1049	0.1641	-	0.8290	-
C*04:01	0.4815	0.4810	-	0.3371	-
C*04:13	0.0154	0.1998	-	0.6601	-
C*05:01	0.0154	0.1492	-	0.2960	-
C*06:02	0.0401	0.6038	-	0.7756	-
C*07:01	0.0895	0.1676	-	0.6181	-
C*07:02	0.1605	0.6792	-	0.7762	-
C*07:18	0.0864	0.7958	-	0.3811	-
C*08:02	0.0648	0.0986	-	0.5319	-
C*14:02	0.0432	0.8562	-	0.5165	-
C*15:05	0.0309	0.5444	-	**0.0362***	0.6511
C*16:01	0.2531	0.6591	-	0.7802	-
C*17:01	0.1975	0.1083	-	0.6509	-
C*18:02	0.0741	0.3865	-	0.0577	-

[a]*P*-value was obtained using Welch’s t-test. *Pcorrected* indicates the corrected *P*-value.
